# Multisensory processing and proprioceptive plasticity during resizing illusions

**DOI:** 10.1007/s00221-023-06759-7

**Published:** 2024-01-02

**Authors:** Kirralise J. Hansford, Daniel H. Baker, Kirsten J. McKenzie, Catherine E. J. Preston

**Affiliations:** 1 University of York, Heslington, York, YO10 5DD UK; 2 University of Lincoln, Brayford Pool, Lincoln, LN6 7TS UK

**Keywords:** Multisensory processing, Resizing illusions, Proprioceptive plasticity, Audition

## Abstract

Bodily resizing illusions typically use visual and/or tactile inputs to produce a vivid experience of one’s body changing size. Naturalistic auditory input (an input that reflects the natural sounds of a stimulus) has been used to increase illusory experience during the rubber hand illusion, whilst non-naturalistic auditory input can influence estimations of finger length. We aimed to use a non-naturalistic auditory input during a hand-based resizing illusion using augmented reality, to assess whether the addition of an auditory input would increase both subjective illusion strength and measures of performance-based tasks. Forty-four participants completed the following three conditions: no finger stretching, finger stretching without tactile feedback and finger stretching with tactile feedback. Half of the participants had an auditory input throughout all the conditions, whilst the other half did not. After each condition, the participants were given one of the following three performance tasks: stimulated (right) hand dot touch task, non-stimulated (left) hand dot touch task, and a ruler judgement task. Dot tasks involved participants reaching for the location of a virtual dot, whereas the ruler task concerned estimates of the participant’s own finger on a ruler whilst the hand was hidden from view. After all trials, the participants completed a questionnaire capturing subjective illusion strength. The addition of auditory input increased subjective illusion strength for manipulations without tactile feedback but not those with tactile feedback. No facilitatory effects of audio were found for any performance task. We conclude that adding auditory input to illusory finger stretching increased subjective illusory experience in the absence of tactile feedback but did not affect performance-based measures.

**Supplementary Information:**

The online version contains supplementary material available at 10.1007/s00221-023-06759-7.

## Introduction

Resizing illusions can be delivered through either augmented reality or magnifying optics and typically use combined visual and tactile inputs to manipulate the size of a body part, making it appear either larger or smaller. These illusions, through changing the way a body part is perceived, are thought to exploit principles of multisensory processing to elicit modulations in the perceived size and shape of the body part (Preston and Newport 2011; Preston et al. 2020; Stanton et al. 2018). In addition to visual and tactile illusions, the combination of visual and proprioceptive, or visual and motor inputs, has also been found to elicit body resizing illusions. Research demonstrates that proprioceptively aligning a child’s avatar body with a participant’s adult body can elicit a strong illusion of having a smaller child-sized body (Banakou et al. 2013). Further research also similarly shows that synchronous movements of an avatar with an elongated arm influence participants’ judgements of arm length (Kilteni et al. 2012). Furthermore, tasks using combined visuotactile inputs have been compared to those employing unimodal visual inputs for finger-stretching illusions, with participants reporting greater subjective embodiment of the illusion during combined visuotactile stimulation than that during unimodal visual illusions (Hansford et al. 2023). Such findings serve to highlight the importance of multisensory processing for subjective embodiment during illusory changes in finger length.

Multisensory processing helps us to perceive a stimulus as a single coherent experience, despite comprising a combination of several different sensory inputs. This process is thought to be important for experiencing our body as our own, as has been demonstrated during the rubber hand illusion, whereby the simultaneous visual and tactile stimulation of a fake hand, at the same time and location as inputs applied to a participant’s own visually occluded hand, can manipulate our understanding of what we experience to be part of our own body (Botvinick and Cohen 1998). Theories explaining body ownership and multisensory body illusions focus primarily on tactile and proprioceptive inputs (Tsakiris 2010; Botvinick and Cohen 1998) as these senses are thought to be unique to bodily experience. Sensory inputs such as vision, which is understood to be weighed heavily in multisensory integration processes during body illusions (Makin et al. 2008), and audition, which is thought to be a more external sense, are experienced both in relation to our own body and to objects in the external world. However, more recent Bayesian accounts of body ownership suggest that the addition of other senses may also facilitate feelings of embodiment and vividness of body illusions (Kilteni et al. 2015). Studies have claimed additive effects of additional senses in multisensory integration concerning non-body events, a finding that the addition of auditory stimuli enhanced overall efficiency in difficult visual detection tasks (Frassinetti et al. 2002). This has also been demonstrated in the other direction, showing that visual cues can aid the detection of low-intensity sounds (Lovelace et al. 2003). In addition, there is evidence supporting the modulation of tactile perception via audio cues; a study by Zampini and Spence (2004) found that increasing the overall volume and/or the amplitude of high-frequency sounds, combined with the tactile input of biting a potato chip, increased the reported crispness of the chip.

Research examining multisensory processing relating to the body and body illusions has also begun to explore the importance of other senses; notably, the role of auditory inputs in multisensory interactions, which have been found to influence perceptions of body size and length (Tajadura-Jiménez et al. 2012), as well as altering perceived material properties (Senna et al. 2014) and the weight (Tajadura-Jiménez et al. 2015a, b) of the body. Looking specifically at visual, tactile and auditory inputs within the rubber hand illusion (which is used as an experimental test for embodiment experienced in resizing illusions), O’Mera (2014) used proprioceptive drift tasks, which measure localisation bias after proprioceptive manipulations, and found that adding auditory inputs consistent with the visual and tactile inputs related to stroking the hand (in this instance, the sound of sandpaper scratching the skin) heightened the illusory experience more than when white noise was added to the illusion. This is further supported by the findings of Radziun and Ehrsson (2018), who also looked at the addition of ecologically relevant auditory inputs to the rubber hand illusion. Their study used the sound of a surface being stroked with a paintbrush, subjective questionnaires and proprioceptive drift tasks to demonstrate that synchronous auditory cues made the illusion stronger, compared to asynchronous auditory cues.

The addition of auditory inputs in the studies mentioned above involved naturalistic auditory inputs, i.e. experimental auditory input that was consistent with realistic auditory stimuli, such as we are used to encountering in everyday life. However, Tajadura-Jiménez et al. (2017) looked at the influence of non-naturalistic auditory inputs, to see whether this still resulted in changes to body perception. Here, they used changes in pitch, due to their associations with a change in height or size (Hubbard 2018), and which are not typically associated with bodily movement. They found that when participants pulled their own right index finger with their left hand, with an accompanying rising pitch sound (700–1200 Hz) and an absence of any visual information, they estimated the length of their index finger to be longer than when this pulling was accompanied with either a descending (700–200 Hz) or constant (700 Hz) tone and coined this the ‘auditory Pinocchio’ effect (although they did not attempt to stretch participants’ noses).

Given these previous findings involving naturalistic auditory inputs in the rubber hand illusion (O’Mera 2014) and non-naturalistic auditory inputs in auditory-tactile resizing manipulations (Tajadura-Jiménez et al. 2017), it is plausible that the addition of non-naturalistic auditory inputs accompanying a visual input of a finger changing size through the use of augmented reality to induce visual and visual-tactile resizing illusions could increase the strength of the illusory experience. This prediction refers again to the notion that the inclusion of more senses provides a more holistic and vivid experience of an event (Kilteni et al. 2015).

Measuring the experience of illusory effects often consists of questionnaires given to participants after they have experienced an illusory condition to gain a subjective measure of their experience. However, more performance-based evidence can also be taken from behavioural measures of proprioceptive drift, which is defined as the change in proprioceptively perceived position of the participant’s hidden body part (Davies et al. 2013). Previous studies assessing proprioceptive drift during the rubber hand illusion have produced conflicting results regarding the influence of the illusion on body schema. Body schema are representations of the body based on bottom-up sensory inputs that are needed for action, and are thought to be distinct from body image, which refers to a top-down body representation that is needed for perception (Paillard 1999). Kammers et al. (2009) investigated the relationship between body schema and body image using the rubber hand illusion with a reaching proprioceptive drift task (action task), wherein participants were asked to reach with one hand to point to the tip of the index finger of the other hand in a single movement, to assess body schema. The participants were also asked to verbally report when the experimenter’s moving finger matched the felt location of their own finger (perceptual task), to assess body image. Kammers et al. found that only the perceptual judgements regarding limb ownership were sensitive to distortion in the rubber hand illusion, concluding that action movements, and therefore body schema, were not affected. In contrast, Newport et al. (2010) used augmented reality and a dot touch proprioceptive drift task with supernumerary limbs to assess body schema using a virtual version of the rubber hand illusion and found that distortions in body schema were apparent, evidenced through pointing errors in the dot touch task that were consistent with the remapped limb position.

A point to note within this previous research is that the terms ‘subjective’ and ‘performance task’ can be used to refer to several concepts in relation to data regarding bodily experience. For the purposes of the current study, the term ‘subjective self-reports’ is used to refer to data collected from self-report questionnaires, whereas the term ‘performance task’ is taken to refer to data collected from proprioceptive plasticity and ruler judgement tasks, such as those used by Davies et al. (2013), Kammers et al. (2009) and Newport et al. (2010). Previous studies concerning the rubber hand illusion typically use proprioceptive drift to assess performance-based illusory experience; however in the current study, we are looking more broadly at proprioceptive plasticity. Proprioceptive plasticity refers to the changeable nature of proprioception that can be influenced by body illusions, but that is not specific to drift from one body part to another such as in the rubber hand illusion. Proprioceptive plasticity acts as a more general term regarding changes to proprioception. This is due to self-report tasks indexing personal, subjective, experience of resizing illusions, whereas proprioceptive drift and ruler judgement tasks index aspects which some researchers consider as more impartial, performance-based, data regarding the effects of resizing illusions on one’s percept of their bodily experience.

Given previous research demonstrating additive effects on the overall illusion experience when including several different sensory inputs, and the recent evidence that additional auditory inputs can affect illusory experience in comparison to unimodal stimulation alone, we hypothesise that through using a between-subjects design wherein one group has non-naturalistic auditory input (one that is consistent with the visual and tactile manipulations of stretching a finger) during augmented-reality resizing illusions, whilst the other group has no auditory input, (1) illusion strength, measured via a subjective illusory experience questionnaire, will be heightened for (1a) visual and (1b) visuotactile manipulations within the audio group. In addition, we hypothesise (2) that the addition of auditory input will lead to stronger illusions as indexed by performance tasks, in line with the experience of a longer finger, as measured using a dot touch proprioceptive plasticity task that indexes body schema for (2a) visual and (2b) visuotactile manipulations. We also hypothesise that the addition of auditory input will increase judgements of finger length, measured using a ruler judgement task that indexes body image for (3a) visual and (3b) visuotactile manipulations. Our inclusion of two different proprioceptive plasticity tasks, a dot touch task and a ruler judgement task, aims to address the apparent discordance between the findings of Kammers et al. (2009) and Newport et al. (2010), relating to the effects of resizing illusions upon body image and body schema.

## Methods

### Pre-registration

Pre-registration of this study can be found at the following OSF link: https://osf.io/6x4ce.

### Ethical approval

This study was granted ethical approval from the University of York.

### Participant sample

#### Power analysis and sample size

*A priori* power analysis using subjective illusion data and performance task dot touch data from a pilot study (*N* = 10, https://osf.io/pb3ku) showed that a minimum sample size of 26 participants is required for hypothesis 1a regarding visuo-auditory/visuotactile-auditory manipulations (Cohen’s *d* = 1.02, power = 0.80, *α* = 0.05, between-subjects design), and a sample of 22 participants is required for hypothesis 2 regarding the dot touch task (*f* = 0.64, power = 0.80, *α* = 0.05, between-subjects design). Due to the inherent ambiguity of effect size estimations used to determine sample sizes in power analysis, and to account for the additional ruler judgement task, the upper sample size of 26 participants was doubled to a sample size of 52 participants.

#### Participants

Fifty-two participants (44 females, 6 males, 2 non-binary; mean age = 19.3 years, age range = 18–24 years, sample population = students at the University of York) gave informed consent, were allocated randomly to either the audio group or the no-audio group, and completed the experiment. A between-subjects design was used to avoid any potential confounding or order effects of the illusions with auditory input. Exclusion criteria were detailed on the participant information sheet and included: prior knowledge or expectations about the research, a history of neurological or psychiatric disorders, any operations or procedures that could damage peripheral nerve pathways in the hands, a history of chronic pain conditions, a history of drug or alcohol abuse, a history of sleep disorders, a history of epilepsy, having visual abnormalities that cannot be corrected optically (i.e. with glasses) or being under 18 years of age. From these 52 participants, 8 scored above 50 (indicating experience of the illusion) on the subjective experience questionnaire item regarding feeling stretching of the finger within the baseline condition where no stretching took place. It was therefore determined that these eight participants did not complete the subjective illusory experience scale correctly, and they have therefore been removed from subsequent analyses, resulting in 44 participants being included in the final sample; 23 in the no-audio group and 21 in the audio group. Analysis of all the 52 participants’ data was completed in line with the pre-registration for transparency and can be seen in Supplementary Materials S9−S11.

### Materials

**Fig. 1:**
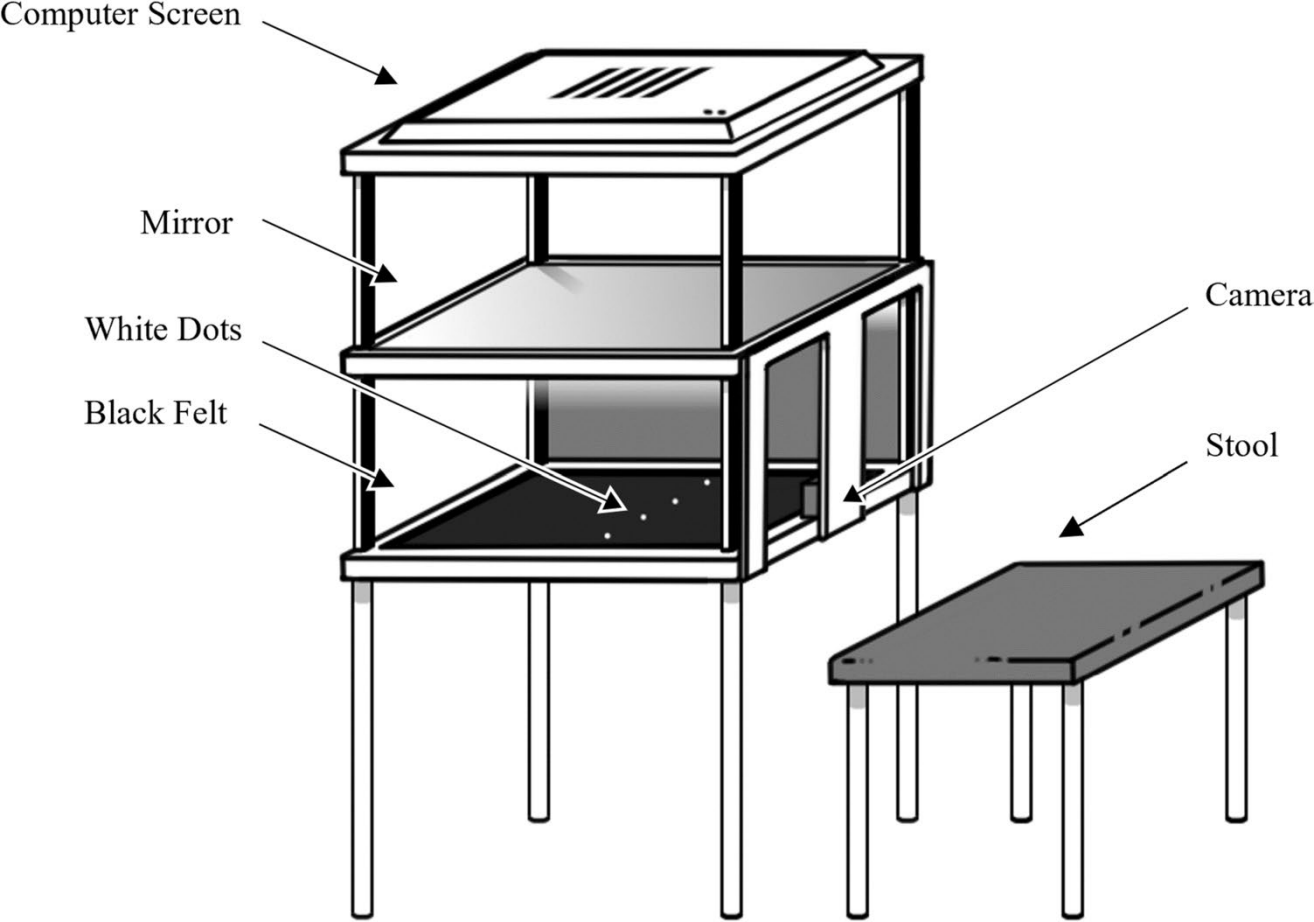
Schematic of augmented reality system

The resizing illusions were delivered using an augmented-reality system (see Fig. 1) that consisted of an area for the hands to be placed which contained a black felt base, LED lights mounted on either side and a 1920 × 1080 camera situated in the middle of the area, away from the participant’s view. Above this area, there was a mirror placed below a 1920 × 1200 resolution screen, so that the footage from the camera was reflected by the mirror such that the participant could view live footage of their own occluded hands. The manipulation of the live feed from the camera was implemented using MATLAB r2017a, wherein the participant’s finger would stretch by 60 pixels (2.1 cm) during illusions lasting 2.4 s. This stretching was accompanied during the visuotactile/visuotactile-auditory conditions by the experimenter gently pulling on the participant’s right index finger to provide tactile input and induce immersive multisensory illusions. In the audio group, the stretching manipulations in the visuotactile-auditory and the visual-auditory conditions were accompanied by a pure tone that increased linearly in frequency from 308 to 629 Hz. Trials during which no stretching took place were accompanied by a 440-Hz tone. Auditory input was delivered by two speakers located beneath the augmented reality system. This positioning of the speakers was to ensure that the location of the sound was aligned with the location of the resizing manipulations (based on feedback from the pilot study that suggested auditory input delivered further from the augmented-reality system created a disconnection between the different sensory inputs). After each condition, the participant’s hands were occluded from view and the dot touch or ruler judgement tasks were presented (detailed in ‘Participant Sample’), until the experimenter pressed a button to indicate the start of the next trial. A blue rectangle was superimposed on the screen so that the participants knew where to reposition their hands to after each task. Subjective illusion experience data were collected via Qualtrics (Qualtrics, Provo, UT) on a Samsung Galaxy Tab A6 tablet. This was given to the participants after all experimental trials were presented, when each manipulation was presented again, without the subsequent tasks, and the participants were asked to recall the trial they had just experienced and previous trials that were similar, and then give a response on a visual analogue scale of 0 to 100, with 0 being strongly disagree, 50 being neutral and 100 being strongly agree, with written statements. The questionnaire consisted of six statements, two relating to illusory experience: ‘It felt like my finger was really stretching’/‘It felt like the hand I saw was part of my body,’ two relating to disownership: ‘It felt like the hand I saw no longer belonged to me’/‘It felt like the hand I saw was no longer part of my body,’ and two were control statements: ‘It felt as if my hand had disappeared’/‘It felt as if I might have had more than one right hand.’ The questionnaire was delivered three times, once after baseline manipulations, once after visuotactile/visuotactile-auditory manipulations and finally once after unimodal visual/visual-auditory manipulations.

### Procedure

The participants were assigned to either the auditory group or the non-auditory group based on a randomised MATLAB output of the total number of participants split randomly and evenly into two groups. They were then seated at the augmented-reality system and were instructed to place both of their hands onto the felt lining, with their index fingers outstretched. There were four white dots on the felt to guide where their hands should be placed, creating two hand spaces (one between each pair of dots), and arm rests were provided for comfort. The participants were instructed to view the image of their hands in the mirror (whilst their real hands were hidden from view) throughout the experiment. They viewed their hands whilst receiving baseline conditions in which no manipulations were applied (with a 440-Hz tone played for auditory group), stretching conditions in which they saw the index finger on their right-hand visually stretch (unimodal visual/visual-auditory conditions with accompanying 308–629 Hz sound for the auditory group) and stretching conditions in which they saw their index finger on their right hand stretch as a researcher gently pulled on the end of their finger simultaneously (visuotactile/visuotactile-auditory conditions with accompanying 308−629 Hz sound for the auditory group).

After viewing the manipulation of their right hand, the participants completed either a left-hand dot touch task, a right-hand dot touch task or a ruler judgement task. The dot touch tasks consisted of the participant’s hands being occluded from view before a magenta dot appeared in front of either their right or left hand, and the participants were then asked to move their index finger in one smooth ballistic pointing movement to touch the dot. When the participants had completed this movement, they were asked to leave their finger in place for a few seconds whilst the experimenter pressed a button to record an image of the hand position through the camera. The participants then returned their hand to the indicated pre-trial position. The ruler judgement task consisted of the participant’s hands being occluded from view before a 14-cm ruler, with 8 marks spaced 2 cm apart, was displayed to the right of the participant’s right hand. The ruler changed in position and scale to avoid trial order bias. The start point of the scale ranged from 10 to 60 (in arbitrary units), and the vertical position of the ruler was jittered using a normal distribution with a mean of 0 and a standard deviation of 40 pixels. The participants were asked to verbally indicate the location on the ruler that corresponded with where they felt the tip of their right (stimulated) index finger was.

The participants completed six repetitions of nine distinct conditions which can be seen in Table 1. A video of a participant undergoing visuotactile stretching can be seen in supplementary material. Conditions were randomised via MATLAB r2017a, and the experimenter was unaware which condition would be presented on a given trial. The experimenter was informed whether to gently pull the index finger or to apply no manipulation via the presentation of a small blue rectangle on the screen, out of the participant’s view. Six repetitions of the nine conditions were presented, followed by a break for the participant to remove their hands from the box and rest, and then the baseline, visuotactile/visuotactile-auditory and the unimodal visual/visual-auditory conditions were presented once in a random order, without any dot touch or ruler judgement tasks, after which the participant completed the subjective illusory experience questionnaire.

**Table 1:**
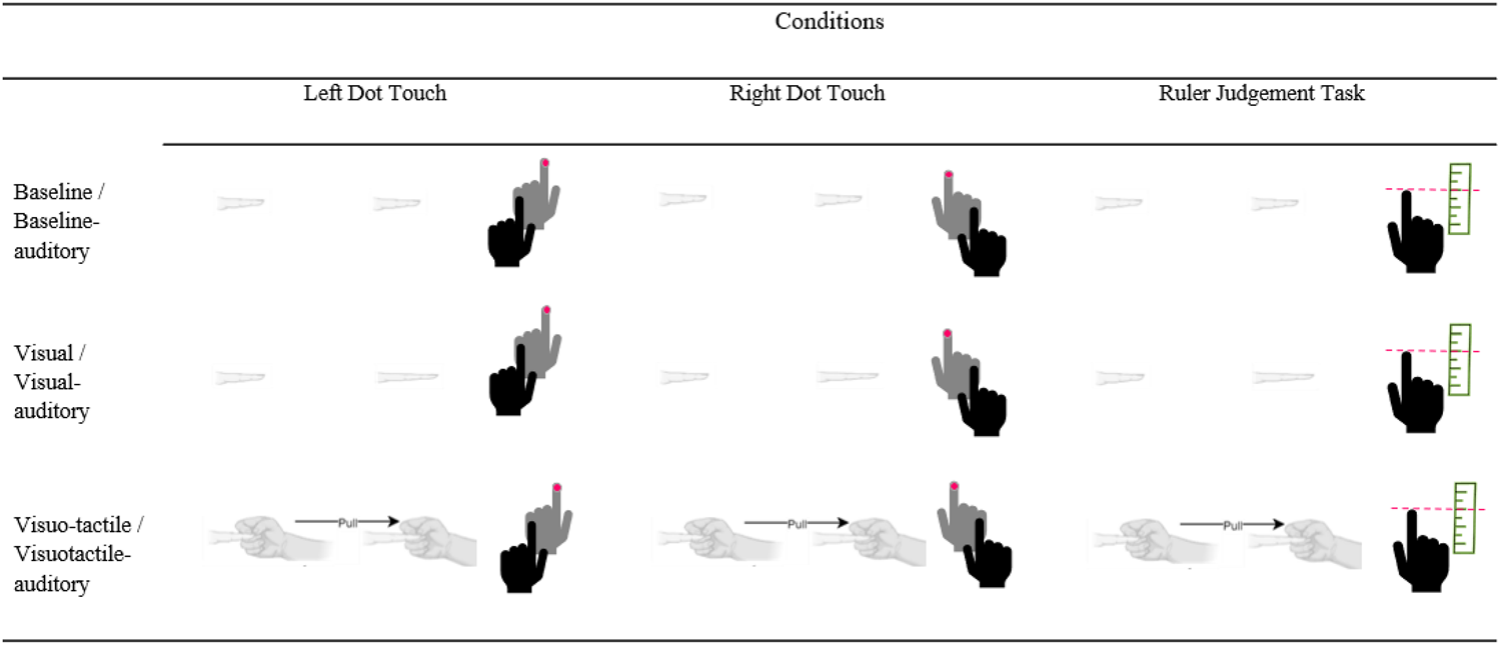
Distinct conditions with associated tasks shown as infographics

The no-audio group experienced the condition without audio, whilst the audio group had auditory input during the resizing illusions (increasing pitch tone) and the baseline trials (constant tone). Performance-based tasks can be seen to the right of each condition under the respective column headers

### Analysis

Questionnaire data were exported from Qualtrics to a .csv file before being loaded into RStudio for analysis.

For the dot touch and ruler judgement data, during each trial, a still image was taken of the location of the participant’s hands within the augmented-reality system. Preprocessing was done algorithmically using image intensity data to estimate finger position; details of this can be seen in the code available on OSF at the following link: https://osf.io/b9s48/. For the dot touch data, the images were used to determine how far away the participant’s finger was from the magenta dot, which was stored as an error rating for each trial and then averaged across the same trial types for each participant. This was completed for both left and right dot touch tasks. The ruler judgement data analysis consisted of using the still images with the superimposed ruler and the ruler ratings given verbally by the participant during the experimental task to check that the rating given was within the range of the ruler. If this was not true, as was the case with four participants, then their data for those trials were removed before statistical analysis (analyses with these participant’s data included can be seen in S11, which shows no deviation from statistical narrative compared to the analyses with these outliers removed). For all the included trials, the differences between the given ruler ratings and the actual tips of the fingers on the still images were used to generate error values, which were then used for statistical analysis.

For statistical analysis of all data, a factorial ANOVA with a within-subjects factor of condition and a between-subjects factor of group were used for hypothesis testing in line with the pre-registration.

All data and code for analysis are available on the OSF page, which also contains resources to computationally reproduce this manuscript, including all analyses, figures and statistical outputs, from the raw data.

## Results

Pre-registration of the study did not account for removal of any participant data; however, eight participants scored above 50 (indicating experience of their finger stretching) in the baseline condition where no stretching was induced. Therefore, it was determined that these participants did not complete the subjective illusory experience scale correctly, and they have been removed from all analyses presented in these results. Full sample analyses (including all participants) can be found in Supplementary Materials S9–S11.

**Fig. 2:**
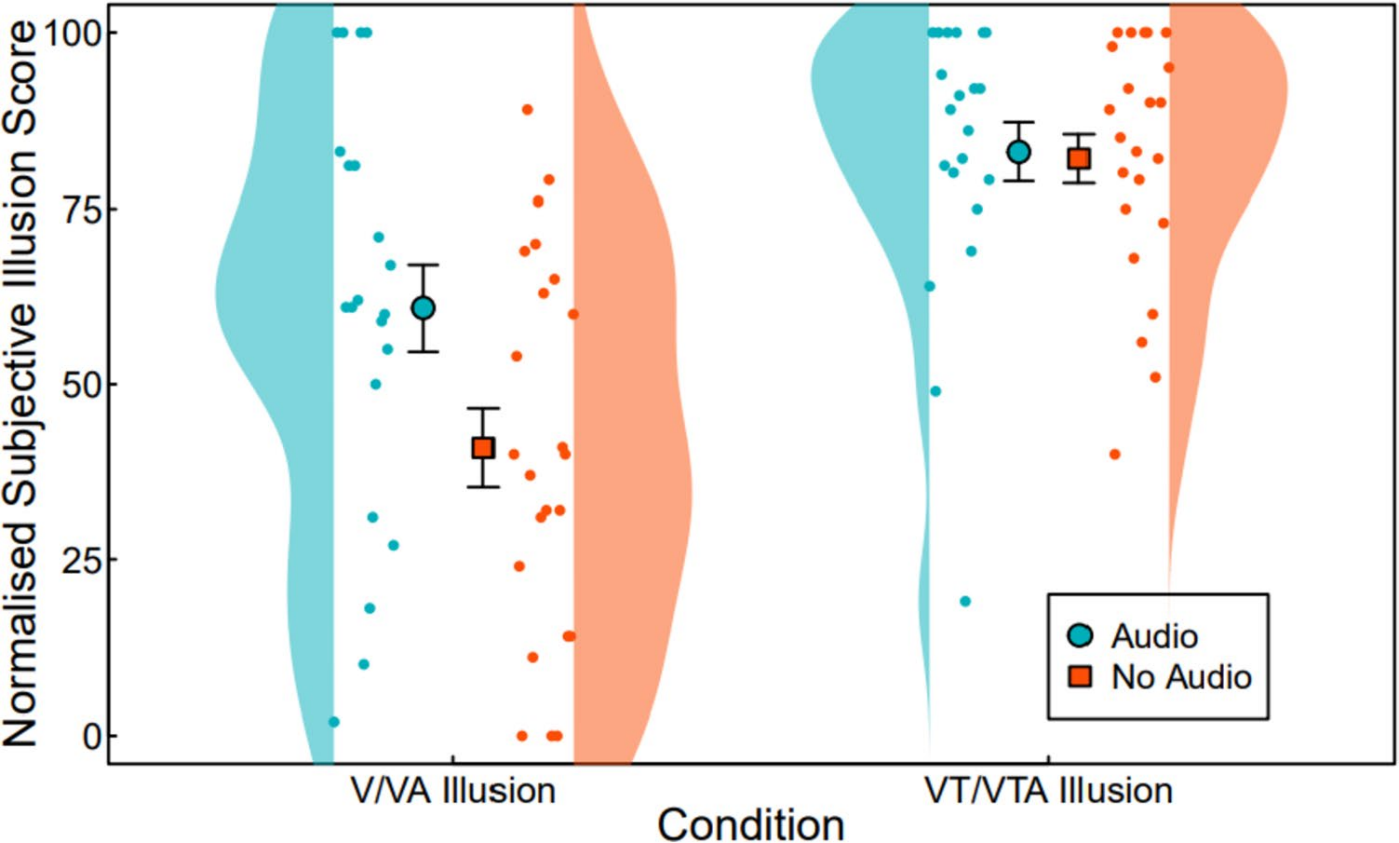
Normalised subjective illusory experience score for V/VA and VT/VTA conditions. Group is indicated by colour, with red showing the no audio group and blue showing the audio group. Error bars show the standard error of the mean, which is shown by the circle and square respectively. Y-axis shows subjective illusion scale data after normalisation through subtraction of each participant’s baseline score from their V/VA and VT/VTA scores. Subjective illusion scale ranges from 0 indicating strongly disagreeing with the experience of finger stretching, 50 indicating a neutral opinion, and 100 indicating strongly agreeing with the experience of finger stretching

Hypothesis 1 predicted that adding a non-naturalistic auditory input to augmented-reality resizing illusions, that is consistent with the visual and tactile manipulations of stretching a finger, would increase subjective illusion strength. We measured this via a subjective illusory experience questionnaire, for (1a) visual and (1b) visuotactile manipulations, with results shown in Fig. 2. The analysis showed a statistically significant interaction between condition and group (*F*(2, 84) = 3.62, *p* = 0.038). Main effects analysis showed that both condition (*F*(2, 84) = 202.31, *p* < 0.001) and group (*F*(1, 42) = 4.48, *p* = 0.04) had a significant effect on subjective illusory experience score. Since a significant interaction was found between condition and group, extending the pre-registered analyses, post hoc pairwise *t* tests with Holm correction for multiple comparisons found that the participants experienced a significantly stronger illusion in the VA condition (*M* = 61.3, SD = 29, SE = 6) compared to the V condition (*M* = 41, SD = 27.1, SE = 6) (*t*(41) = 2.40, *p* = 0.021, CI [−3.39, 29.29]) and found no difference in illusion strength when comparing the VT/VTA conditions (*t*(40) = 0.23, *p* = 0.82, CI [−8.56, 11.09]), indicating that the addition of non-naturalistic auditory input significantly affected subjective illusory experience in the unimodal visual condition, but had no effect on the combined visuotactile condition. In addition, the combination of visual and tactile inputs in the VT condition resulted in a significantly higher mean subjective illusion score (*t*(42) = −2.92, *p* = 0.006, CI [−39.13,−10.76]) (*M* = 82, SD = 17.3, SE = 4) than that in the visual-auditory conditions (*M* = 61.3, SD = 29, SE = 6).

Mean scores across the participants were above 50 (the neutral point of the scale) in all conditions for the second item on the subjective questionnaire, indicating experience of ownership of the seen hand in all conditions, whilst the mean scores for disownership and control statements were below 50, indicating no average disownership of the hand and no average violations of the control statements (results can be seen in Supplementary Materials S2–S4).

Positive control analyses were run on the performance data to check that we were able to see an effect of the illusion with the dot touch and ruler judgement tasks. Positive control data plots can be seen in Supplementary Materials S5−S7. For the right dot touch data, we found a significant effect of condition (*F*(2, 84) = 31.25, *p* < 0.001). Post hoc tests for multiple pairwise comparisons found that the participants placed their finger significantly lower than the dot in the V/VA condition (*p* < 0.001, *M* = −0.89, SD = 1.27, SE = 0.19, CI[−0.90, 0.6) and the VT/VTA condition (*p* < 0.001, *M* = –1.07, SD = 1.19, SE = 0.18, CI [–0.90, 0.60]) compared to the baseline condition (*M* = –0.21, SD = 1.14, SE = 0.17), indicating that an effect of the finger-stretching manipulation was indexed by this performance measure; the participants experienced their index finger as significantly longer under these manipulation conditions and this subsequently produced a measurable effect upon body schema. For the left dot touch data, we found a significant effect of condition (*F*(2, 84) = 18.345, *p* < 0.001). Post hoc tests for multiple comparisons found that the participants placed their finger significantly lower than the dot in the V/VA condition (*p* < 0.001, *M* = –1.26, SD = 1.44, SE = 0.22, CI [–0.48, 1.27]) and the VT/VTA condition (*p* = 0.009, *M* = –0.89, SD = 1.29, SE = 0.19, CI [–0.43, 1.14]) compared to the baseline condition (*M* = –0.63, SD = 1.2, SE = 0.18). Finally, for the ruler judgement data, we found a significant effect of condition (*F*(2, 84) = 11.5, *p* < 0.001). Post hoc tests for multiple pairwise comparisons found that the participants judged their finger to be significantly longer in the V/VA condition (*p* < 0.001, *M* = –0.81, SD = 1.69, SE = 0.26, CI [–1.79, 0.23]) and the VT/VTA condition (*p* = 0.006, *M* = –0.91, SD = 0.28, SE = 0.28, CI[–1.99, 0.26]) compared to the baseline condition (*M* = –1.35, SD = 1.52, SE = 0.23).

**Fig. 3:**
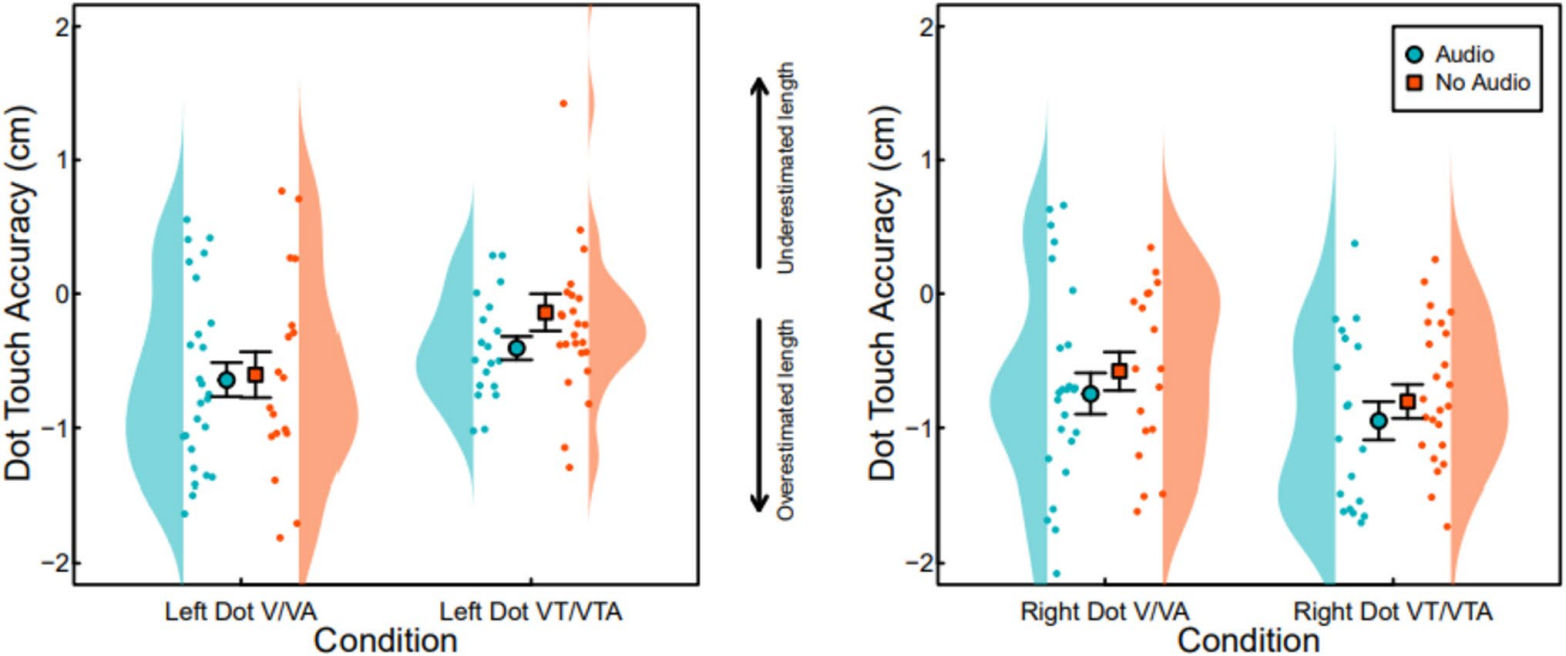
Dot touch data in centimetres for V/VA and VT/VTA conditions for both left and right hand data, relative to baseline judgements. Group is indicated by colour, with red showing the no audio group and blue showing the audio group. Arrows denote the direction of finger length estimation (downward arrow showing overestimation, upward arrow showing underestimation)

We then addressed hypothesis 2 that the addition of auditory input would lead to stronger illusions as indexed by performance tasks in line with the experience of a longer finger, using a dot touch proprioceptive drift task as an index of body schema for (2a) visual and (2b) visuotactile manipulations (see Fig. 3). Analysis of right dot touch data (Fig. 3b) showed no significant interaction between condition and group (*F*(1, 42) = 0.75, *p* = 0.391), and the main effects showed no effect of condition (*F*(1,42) = 2.11, *p* = 0.154) or group (*F*(1,42) = 0, *p* = 0.971). Analysis of left dot touch data (Fig. 3a) showed no significant interaction between condition and group (F(1, 42) = 0.43, *p* = 0.516), whilst the main effects showed no effect of group (*F*(1,42) = 0.03, *p* = 0.858) but did show an effect of condition (*F*(1,41) = 11.09, *p* = 0.002, CI [–0.65,–0.09]), with participants placing their finger significantly lower in the V/VA condition (*M* = –0.63, SD = 0.69, SE = 0.1) compared to the VT/VTA condition (*M* = –0.26, SD = 0.62, SE = 0.09), indicating that the participants experienced a longer finger in the V/VA condition compared to the VT/VTA condition.

**Fig. 4:**
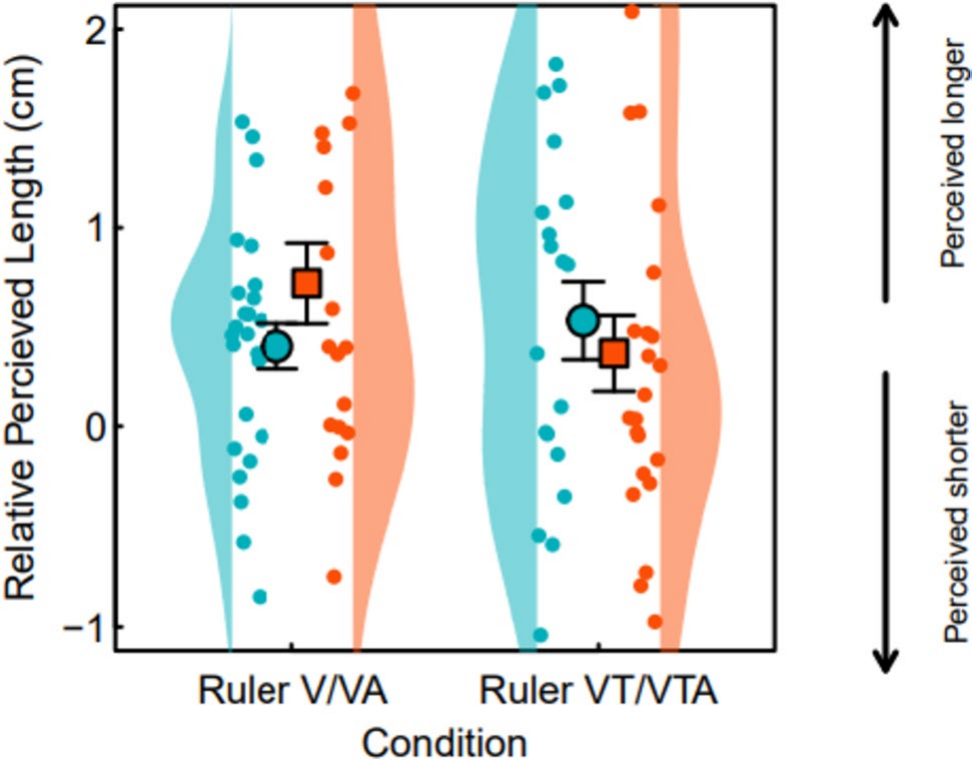
Ruler judgement data in relative centimetres for V/VA and VT/VTA conditions. Group is indicated by colour, with red showing the no audio group and blue showing the audio group. Arrows denote direction of perceived finger length (downward arrow showing shorter perception, upward arrow showing longer perception)

Finally, we assessed hypothesis 3 that the addition of auditory input would heighten ability, measured as differences between reported finger length and actual finger length, on a performance task using a ruler judgement task that indexes body image for (3a) visual and (3b) visuotactile manipulations (see Fig. 4). The analysis showed no significant interaction between condition and group (*F*(1, 42) = 0.334, *p* = 0.567), and the main effects showed no effect of condition (*p* = 0.336) or group (*p* = 0.639).

In addition to analyses planned within our pre-registration, at the suggestion of a reviewer, exploratory correlation analyses were run to assess relationships between subjective illusion score and performance-based measures of resizing illusions. We found no significant relationships between subjective illusion score and performance on any task, under any condition. Further details can be seen in Supplementary Material S11.

## Discussion

This study sought to understand what impact the addition of non-naturalistic auditory input would have on traditional visuotactile and unimodal visual hand-based resizing illusions. Our results showed that the addition of non-naturalistic auditory input, that was consistent with the resizing illusion, increased subjective experience of the illusion in the traditional unimodal visual condition, with participants experiencing a significantly stronger illusion in the visual-auditory condition as compared to the visual-only condition, supporting our first hypothesis. However, we found no facilitatory effects of auditory input for subjective experience of illusion strength within the combined visuotactile condition, or for either of the performance tasks, which was in opposition to our remaining hypotheses and served to highlight a potential discordance between the conscious subjective experience of resizing illusions compared to more unconscious performance-based responses. This discordance was reinforced by exploratory correlation analyses showing no significant relationships between subjective illusion scores and either performance-based task.

The subjective findings showed that participants in the audio group rated their experience of the illusion to be greater in the visual condition compared to the non-audio group, showing that the suggested effects of multisensory processing might be heightening the experience of a stimulus. There was, however, no difference between the audio group and the non-audio group in the visuotactile condition, likely due to ceiling effects, wherein the addition of auditory input to visuotactile input did not increase subjective experience of the illusion. The combination of visual and tactile inputs resulted in a significantly higher mean subjective illusion score than that in the visual-auditory conditions, demonstrating that the combination of two different senses produces differing levels of subjective experience of the illusion, with visuotactile surpassing that of visual-auditory manipulations. It is likely that this increased subjective experience within the visuotactile condition is due to the specific nature of the tactile and proprioceptive inputs, which are thought to be specific to the bodily experience, whereas senses such as vision and audition are experienced not only in relation to our body but also relating to objects in the external world (Tsakiris 2010; Botvinick and Cohen 1998). Therefore, it is plausible that including a sense that is integral to our bodily experience, such as a tactile input, would have a greater effect on body illusions in comparison to less embodied senses such as an auditory input. This is supported by Ernst and Banks (2002), who proposed the theory that sensory inputs are combined in a statistically optimal fashion based on their reliability in reflecting the accuracy of a given stimulus. In the current resizing illusions, Ernst and Banks’ theory explains our findings of a greater illusory experience in the visuotactile condition compared to visual-auditory condition, since the tactile input was more task relevant and came from the same perceived spatial location as the visual input, resulting in the tactile input being upweighted, and therefore had a greater influence on the combined illusory percept, whereas the auditory input was comparatively downweighted. However, when there is an absence of a tactile input, such as in the visual-auditory condition, then the temporal synchrony of the auditory input and visual input serves to upweight the auditory input, allowing a greater influence on the combined percept within the resizing illusion.

Regarding performance findings, our positive control analyses showed that there was a significant difference between baseline and experimental conditions for left and right dot touch tasks, with participants accurately placing their finger on the dot in the baseline condition for the right dot task, and then touching around a centimetre too close to their own bodies in both experimental conditions due to the perceived elongation of the finger in the experimental conditions. For the left dot touch data, the participants were less accurate in their finger placement in the baseline condition, but still placed their finger significantly closer to their own bodies in both experimental conditions, indicating a perceived elongation of their finger in both right and left dot touch tasks. In addition, in the ruler judgement task, the participants reported the tip of their finger to be significantly further away in both experimental conditions compared to the baseline condition. This indicates that they experienced their finger as being longer in both experimental conditions when compared to the baseline non-illusion condition. These findings indicate success of the positive control analyses, showing that these performance tasks can highlight the differences between baseline and experimental conditions.

Referring to the confirmatory analyses regarding the dot touch data, our findings showed no significant effect of group or condition for the right dot touch task, and there was no effect of group for the left dot touch task, however there was an effect of condition, with participants placing their finger significantly closer to their bodies in the conditions without touch (V/VA) compared to the conditions with tactile input (VT/VTA). This finding of a significant effect of condition for the left dot touch data could be explained as a transference effect of stretching from the manipulated hand (right) to the non-manipulated hand (left). Petkova et al. (2011) found whilst using a full body illusion and fMRI evidence for a spread of ownership across connected body parts. Therefore, the resizing of the right hand could likely spread to the left unmanipulated hand, meaning participants felt as though this hand had also been resized, which is supported by the positive control analyses for the left dot touch task in which we found a significant effect of the illusion in the experimental conditions without manipulation of this hand. It is possible that the tactile inputs in the VT/VTA illusion could provide a grounding effect, wherein the participant’s hand is grounded to the spatial location within the augmented-reality system, which does not occur for visual-only or visual audio manipulations. This is further supported by Ernst and Banks’ (2002) optimal integration model, with the tactile input providing a more accurate location estimate than the visual input alone, as the visual input is less reliable than the tactile input and is therefore downweighted in comparison to the tactile input which is upweighted within the combined percept. This spatial grounding in the tactile input conditions in conjunction with the transference effects mentioned previously could explain why we see a significant difference between experimental conditions in the left dot touch task. This is, however, speculative, and further research would be needed to assess the replicability of this effect. Finally, our ruler judgement data also showed no significant effect of condition or group, indicating that the addition of non-naturalistic auditory input showed no facilitatory effects for either performance task.

Exploratory correlation analyses found no significant relationships that survived Bonferroni corrections, thereby reinforcing the discordance observed between our confirmatory findings of a significant effect of group and condition for subjective measures, in comparison to that lack of significant effects for performance-based measures of resizing illusions. The data do, however, show trends towards relationships in the right dot touch and ruler judgement data in relation to subjective illusion score. It is possible that the current study was underpowered to find significant effects in correlation analyses since these analyses were exploratory; therefore, further research is needed to understand the relationship between subjective and performance-based measures of resizing illusions.

The rationale for including two performance tasks in the present study came from previous discordance in the literature with Kammers et al. (2009) finding an impact on body image, but not body schema, with the rubber hand illusion, whereas Newport et al. (2010) found distortions in body schema using the rubber hand illusion and supernumerary limbs. The use of differing measures of body representation in the previous literature often results in different findings, and this discordance between body image and body schema is one example of when this occurs regarding body illusions. Here, we see evidence for an impact of resizing illusions on both body image and body schema, as demonstrated by the positive control analyses, showing that resizing illusions affect one’s percept of the body (body image) in addition to the control of the body in an external environment (body schema). The rubber hand illusion differs from the resizing illusion used here, in that the present manipulation does not attempt to relocate the hand, but rather attempts to alter the representation of the finger to be longer. Therefore, it could be that when changing an existing part of one’s body, both body image and body schema are affected, whilst when attempting to create a new sensation of one’s body in a different location, impact on body schema is dependent on the experimental manipulations being used. In addition, in the current study, we use an augmented-reality system that is similar to that used by Newport et al. (2010), and this system could be producing a more vivid illusion than the rubber hand illusion typically creates.

The increasing pitch tone that was used as the non-naturalistic auditory input in the current study was chosen as it closely reflected that used by Tajadura-Jiménez et al. (2017), who previously found increases in estimations of finger length when they were accompanied by an increasing pitch tone, compared to a decreasing or constant tone. However, in the current experiment, we cannot claim that the effect of an increase in subjective experience of the resizing illusion when this non-naturalistic auditory input is added is unique to a rising pitch tone. It is possible that other auditory inputs could elicit similar effects in increasing subjective experience. Examples might include naturalistic inputs, perhaps of the bones in the finger creaking as it is stretched, akin to the auditory inputs heard during chiropractic treatments, or an unrelated auditory input, such as a constant tone during the resizing conditions. It is also possible that the increasing pitch tone that was used in the current study could be manipulated to be presented in steps, rather than as a constant tone, to assess whether the same effects of increasing illusory experience are seen in different presentations of a rising pitch tone, or whether the addition of any tone at all would increase subjective illusory experience by directing attention towards the illusory manipulation. Nevertheless, the findings from the current study enhance our understanding of the role that auditory input can play in resizing illusions, and further research into the efficacy of alternate auditory inputs should be conducted to consolidate current findings.

Looking into the clinical applications of resizing illusions, it has been suggested that in individuals with chronic pain there may be a cortical misrepresentation of the body and its incoming somatosensory signals, including pain, along with perceptual size dysfunctions of affected limbs, which underpin their persistent pain (Boesch et al. 2016). Since resizing illusions are thought to change one’s representation of their body parts, they have been used within chronic pain populations and have been found to reduce subjective pain ratings in participants with chronic pain conditions affecting the hands (Preston and Newport 2011), back (Diers et al. 2013) and knees (Stanton et al. 2018). The findings from the present study serve to enhance our understanding of the conditions under which these manipulations can affect the personal experience of such illusions. Previously, we have demonstrated that around 30% of participants experience effective resizing illusions via a unimodal visual condition (Hansford et al. 2023). Here, we show that subjective illusion strength during the unimodal visual presentation of finger stretching can be increased through the addition of a simultaneous non-naturalistic auditory input. It is, therefore, possible that when using these resizing illusions for the treatment of chronic pain, it may be beneficial to include non-naturalistic auditory input to increase the subjective illusion strength for patients during the illusion, and consequently, potentially increase attenuation of pain. The unimodal visual condition has been suggested as the most accessible version of resizing illusions (Hansford et al. 2023) as it has the potential to be delivered via a mobile phone application without the need for a researcher to add tactile inputs to the illusion. The incorporation of auditory inputs would not require the presence of a researcher either and, therefore, is a potential method to utilise multisensory integrative processing effects during the unimodal visual application of these illusions to increase subjective illusion strength, which could in turn increase the analgesic effect of these illusions in a chronic pain sample. Future research should, therefore, assess whether the addition of an auditory input has a similar effect in enhancing the strength of these illusions in chronic pain patients, as has been demonstrated here in participants who do not experience chronic pain.

## Conclusion

We found that the addition of non-naturalistic auditory input can increase the subjective illusion strength of resizing illusions administered via a visual input; however, we found no facilitatory effects of the auditory input for any performance measures of illusion strength. We address the previous discordance in the literature surrounding the impact of hand-based illusions on body image and body schema, showing that in a hand-based resizing illusion, the manipulation affects both representations of the bodily self. In addition, this study extends upon previous research finding additive effects of auditory inputs to tactile manipulations of finger resizing and highlights the potential for non-naturalistic auditory inputs to be included in resizing illusions used to treat chronic pain whilst inviting further research to assess the impact of non-naturalistic auditory inputs in chronic pain patient samples. In addition, our findings invite further research into the uniqueness of a rising pitch tone as the presentation of a non-naturalistic auditory input, to assess whether this alone causes an increase in subjective illusion strength. Finally, we highlight the differential effects of these resizing illusions on conscious subjective experience versus unconscious performance-based measures, further elucidating the mechanisms by which such manipulations can alter bodily experience.

### Supplementary Information

Below is the link to the electronic supplementary material.

Supplementary file1 (DOCX 3929 KB)

## Data Availability

All data and materials can be accessed from the following OSF link: https://osf.io/b9s48/.
