# Supplementary material for: Multisensory processing and proprioceptive plasticity during resizing illusions
Source: Exp Brain Res. 2024 Jan 2;242(2):451–62. doi: 10.1007/s00221-023-06759-7 (PMC10805803; doi:10.1007/s00221-023-06759-7)
Supplement: Supplementary file 1 — Supplementary file1 (DOCX 3929 KB) [file 221_2023_6759_MOESM1_ESM.docx]

Supplementary Materials

S1. Video of finger stretching

A video of a participant undergoing a visual-tactile illusion can be seen at the following OSF link: <https://osf.io/ek8cd>


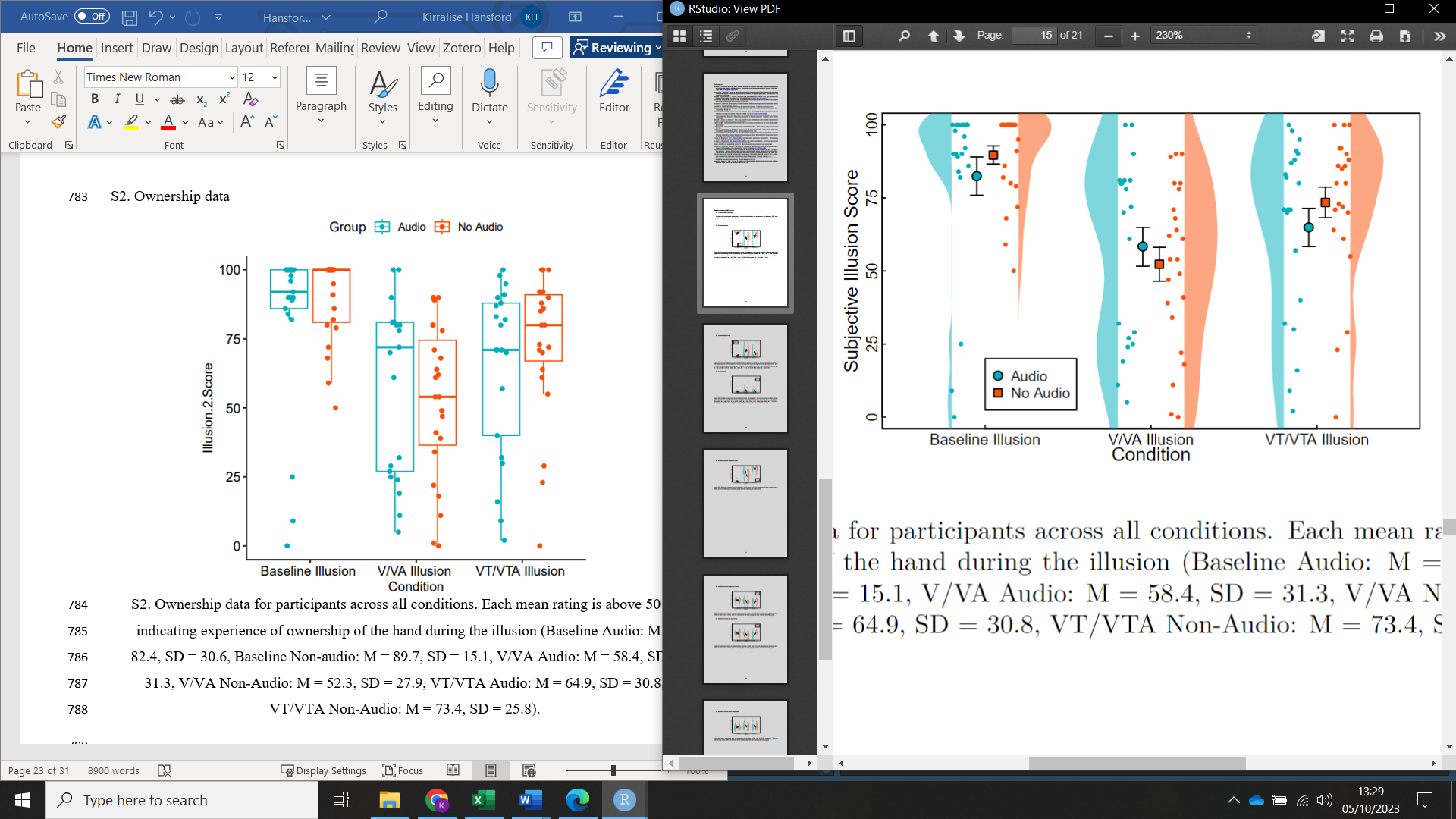
S2. Ownership data

S2. Ownership data for participants across all conditions. Each mean rating is above 50 indicating experience of ownership of the hand during the illusion (Baseline Audio: M = 82.4, SD = 30.6, Baseline Non-audio: M = 89.7, SD = 15.1, V/VA Audio: M = 58.4, SD = 31.3, V/VA Non-Audio: M = 52.3, SD = 27.9, VT/VTA Audio: M = 64.9, SD = 30.8, VT/VTA Non-Audio: M = 73.4, SD = 25.8).


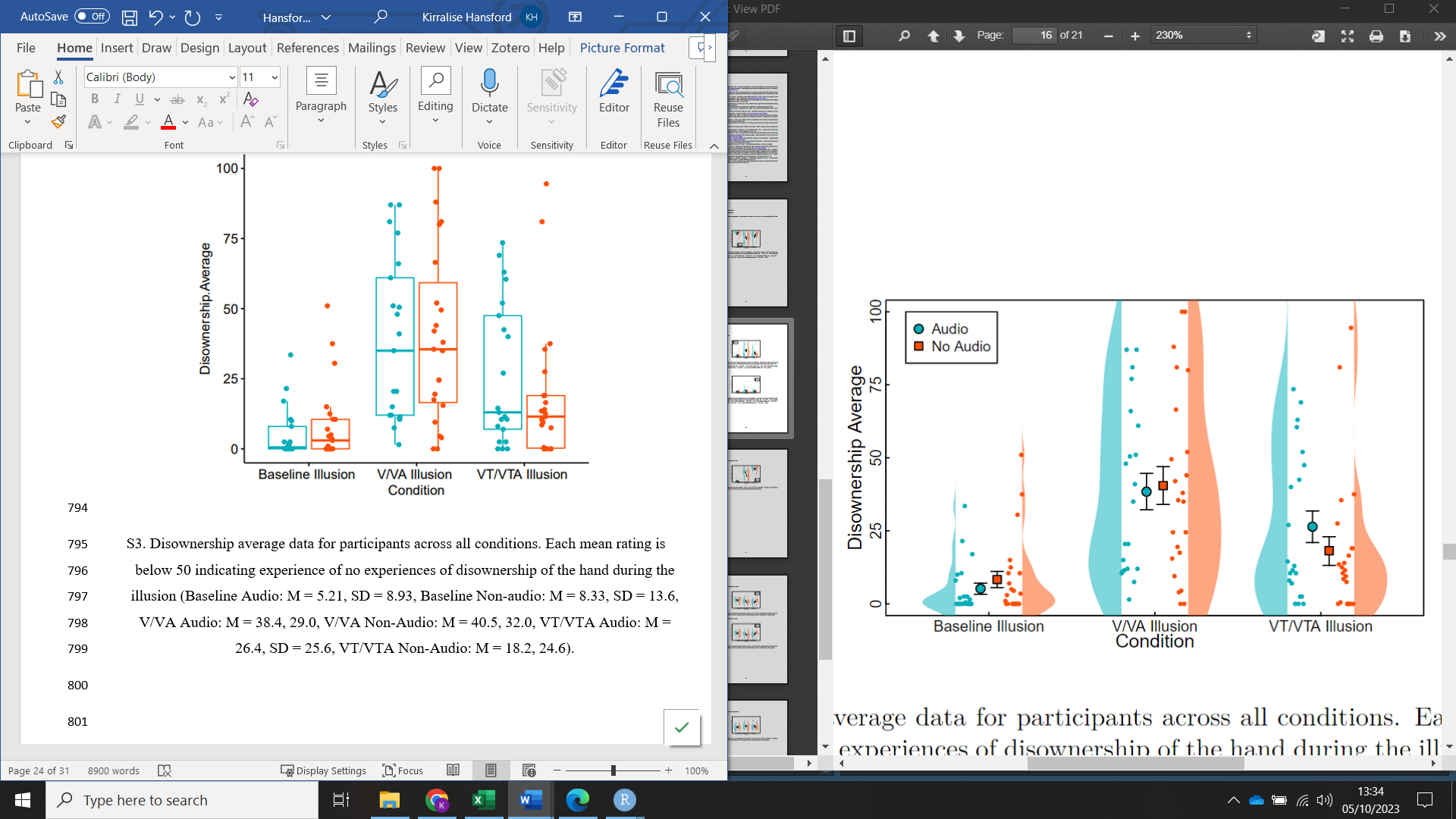
S3. Disownership Data

S3. Disownership average data for participants across all conditions. Each mean rating is below 50 indicating experience of no experiences of disownership of the hand during the illusion (Baseline Audio: M = 5.21, SD = 8.93, Baseline Non-audio: M = 8.33, SD = 13.6, V/VA Audio: M = 38.4, 29.0, V/VA Non-Audio: M = 40.5, 32.0, VT/VTA Audio: M = 26.4, SD = 25.6, VT/VTA Non-Audio: M = 18.2, 24.6).


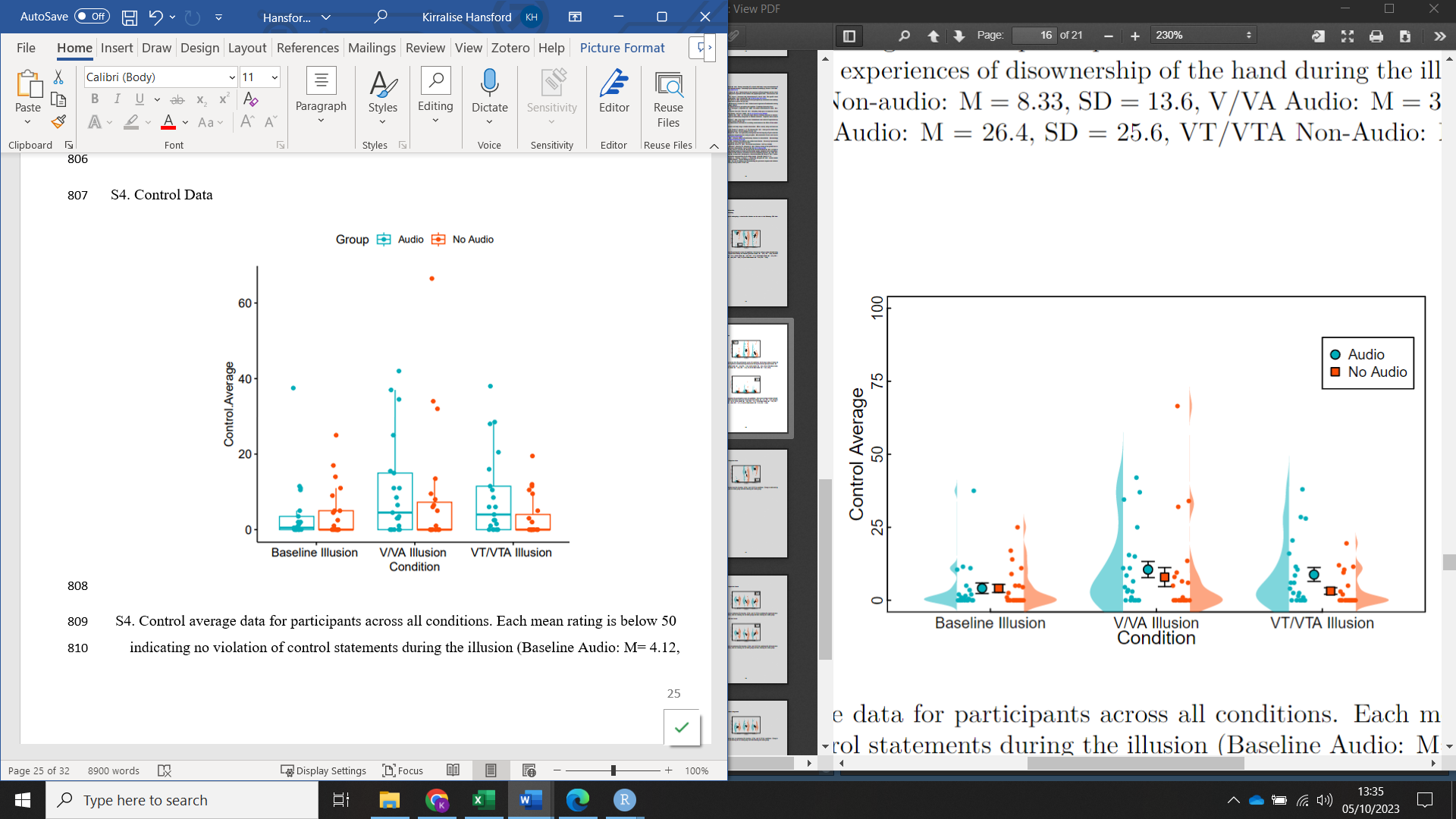
S4. Control Data

S4. Control average data for participants across all conditions. Each mean rating is below 50 indicating no violation of control statements during the illusion (Baseline Audio: M= 4.12, SD = 8.55, Baseline Non-audio: M = 4.09, SD = 6.76, V/VA Audio: M = 10.5, SD = 13.2, V/VA Non-Audio: M = 7.91, SD = 16.0, VT/VTA Audio: M = 8.81, SD = 11.2, VT/VTA Non-Audio: M = 3.17, SD = 5.49).


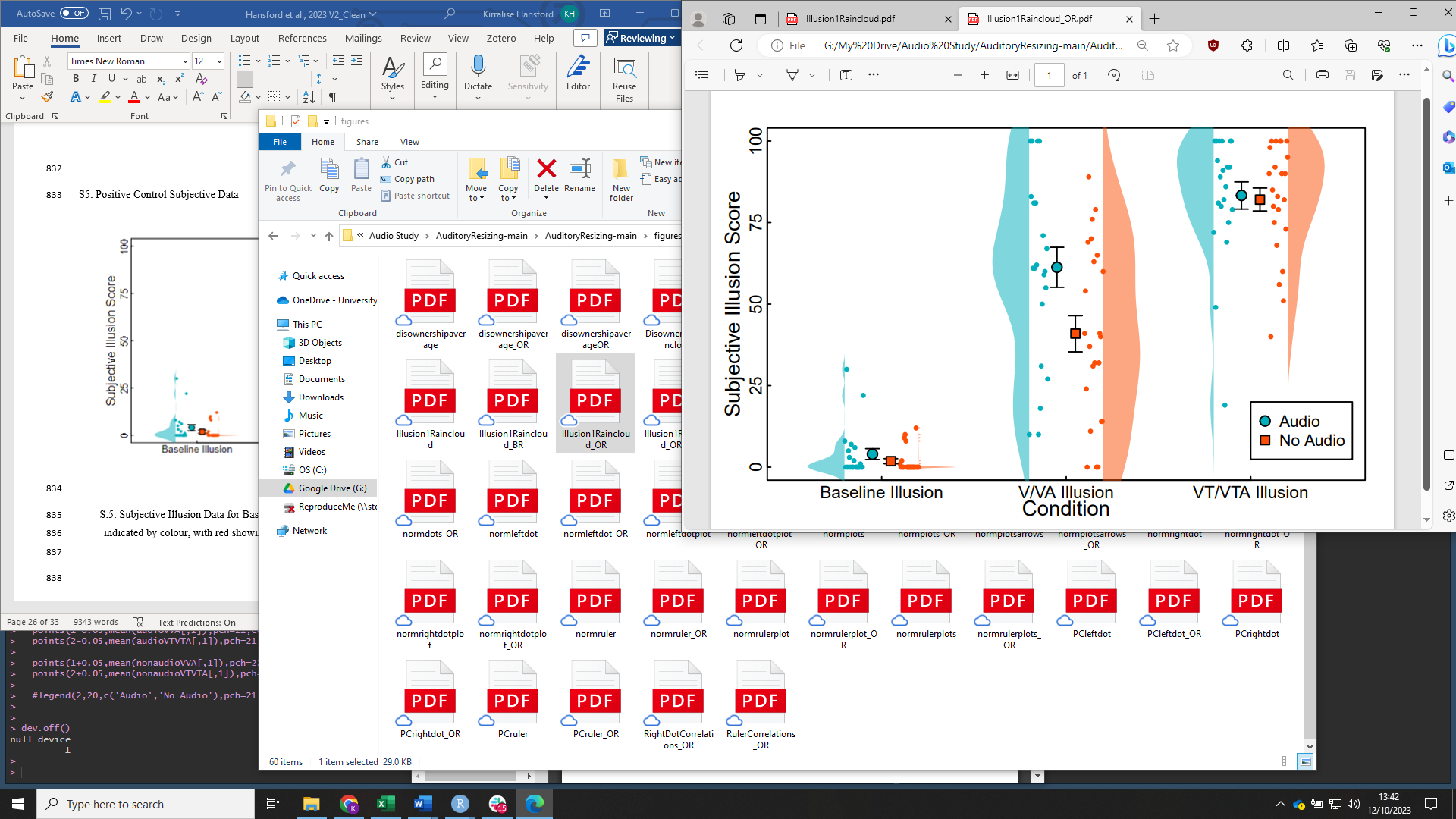
S5. Positive Control Subjective Data

S.5. Subjective Illusion Data for Baseline, V/VA, and VT/VTA conditions. Group is indicated by colour, with red showing the no audio group and blue showing the audio group.


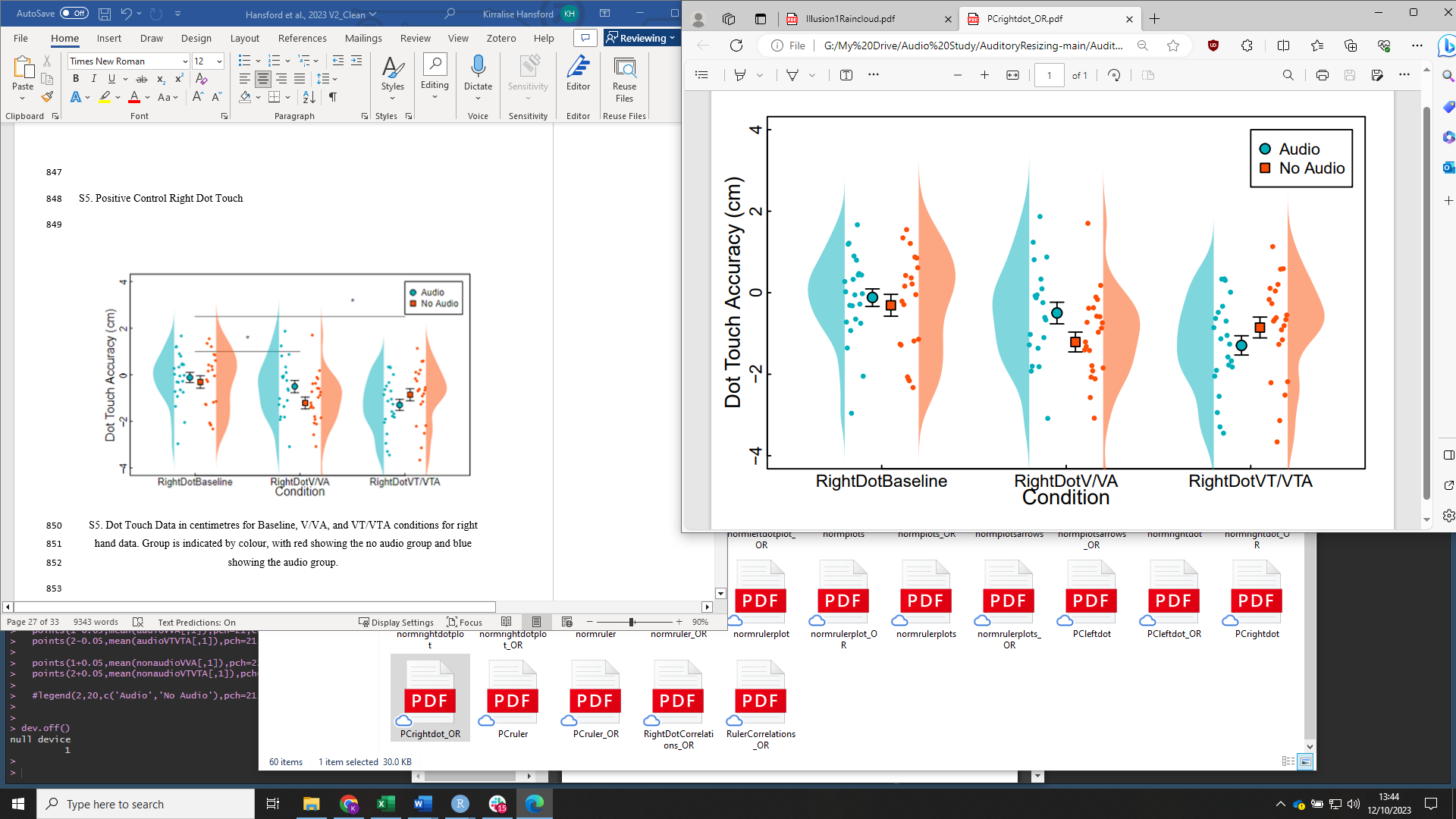
S6. Positive Control Right Dot Touch

S6. Dot Touch Data in centimetres for Baseline, V/VA, and VT/VTA conditions for right hand data. Group is indicated by colour, with red showing the no audio group and blue showing the audio group.


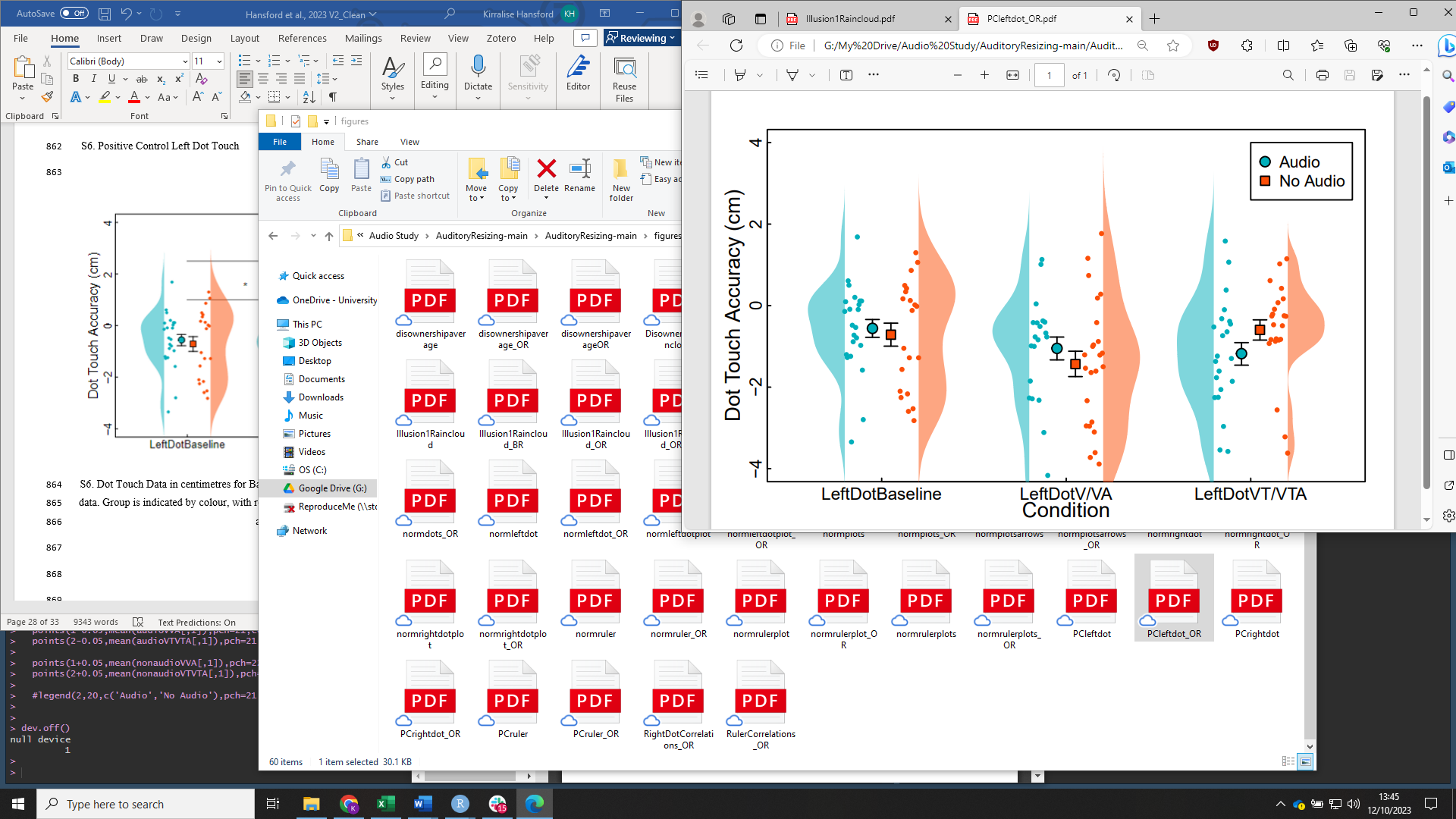
 S7. Positive Control Left Dot Touch

S7. Dot Touch Data in centimetres for Baseline, V/VA, and VT/VTA conditions for left hand data. Group is indicated by colour, with red showing the no audio group and blue showing the audio group.

S8. Positive Control Ruler Judgement


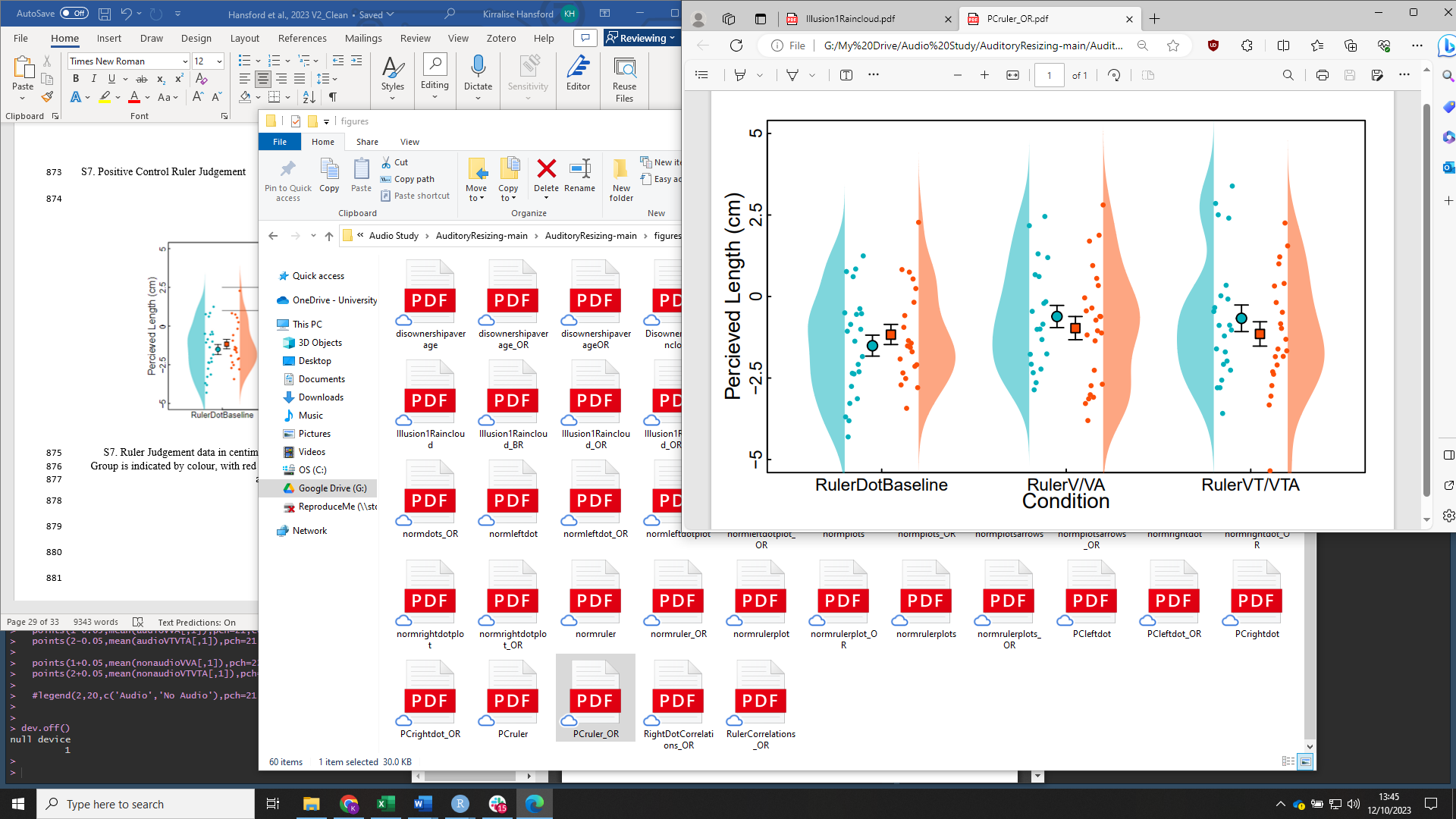


S8. Ruler Judgement data in centimetres for baseline, V/VA, and VT/VTA conditions. Group is indicated by colour, with red showing the no audio group and blue showing the audio group.

S9. Full Sample Subjective Data


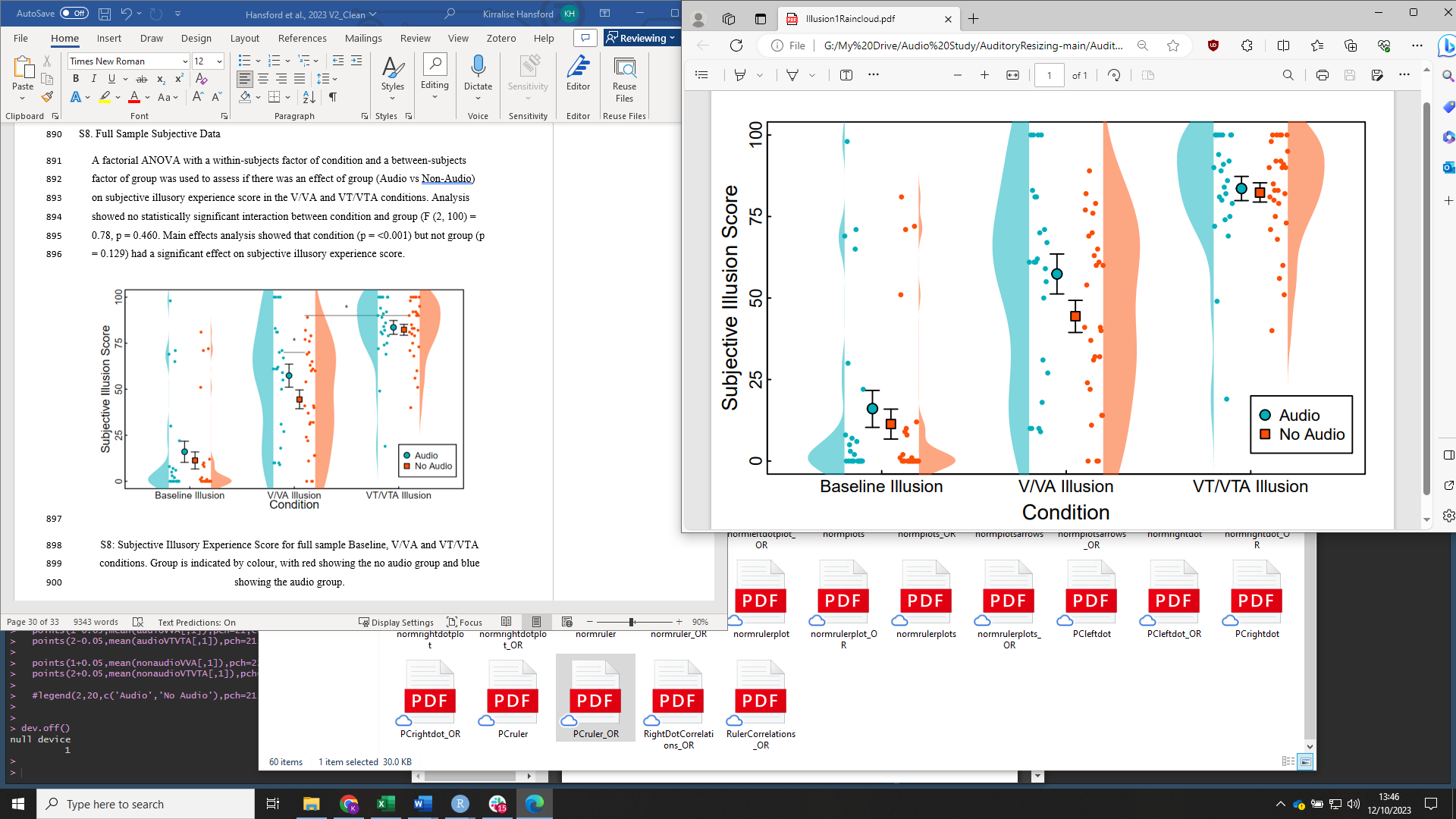
A factorial ANOVA with a within-subjects factor of condition and a between-subjects factor of group was used to assess if there was an effect of group (Audio vs Non-Audio) on subjective illusory experience score in the V/VA and VT/VTA conditions. Analysis showed no statistically significant interaction between condition and group (F (2, 100) = 0.78, p = 0.460. Main effects analysis showed that condition (p = <0.001) but not group (p = 0.129) had a significant effect on subjective illusory experience score.

S9: Subjective Illusory Experience Score for full sample Baseline, V/VA and VT/VTA conditions. Group is indicated by colour, with red showing the no audio group and blue showing the audio group.

S10. Full Sample Dot Touch Data


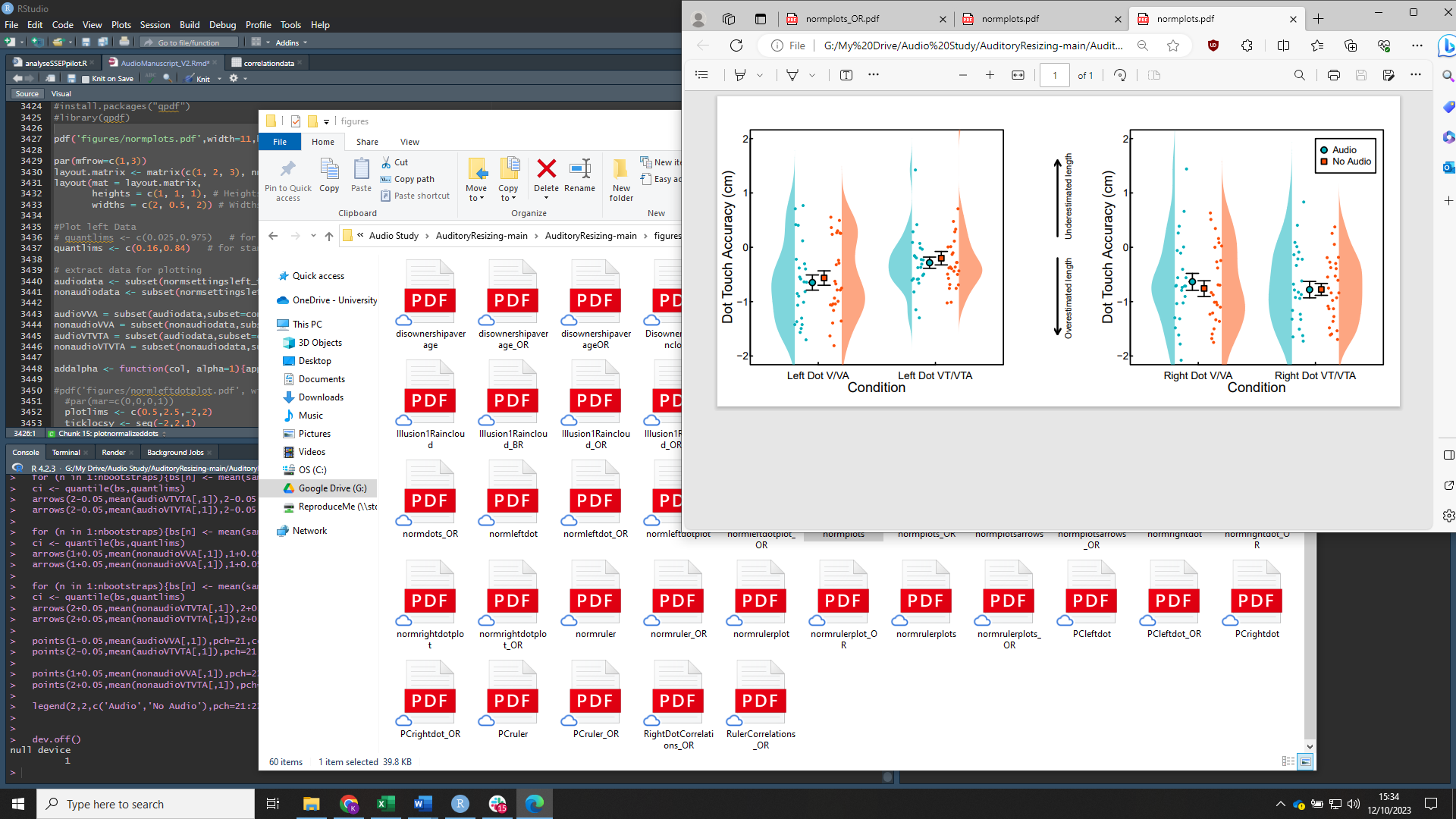
A factorial ANOVA with a within-subjects factor of condition and a between-subjects factor of group was used to assess if there was an effect of group (Audio Vs Non-Audio) on dot touch data in the V/VA and VT/VTA conditions. Analysis on right dot touch data showed no significant interaction between condition and group F(1, 50) = 0.24, p = 0.628 and main effects showed no effect of condition (p = 0.524) or group (p = 0.707). Analysis on left dot touch data showed no significant interaction between condition and group F(1, 50) = 0, p = 0.992 whilst main effects showed no effect of group (p = 0.581) but did show an effect of condition (p = <0.001), with participants placing their finger significantly lower in the V/VA condition (M =-0.6, SD =0.7) compared to the VT/VTA condition (M =-0.24, SD =0.6).

S10: Dot Touch Data in relative centimetres for full sample V/VA and VT/VTA conditions for both left and right hand data. Group is indicated by colour, with red showing the no audio group and blue showing the audio group. Arrows denote the direction of finger length estimation.

S11. Full Sample Ruler Judgement Data


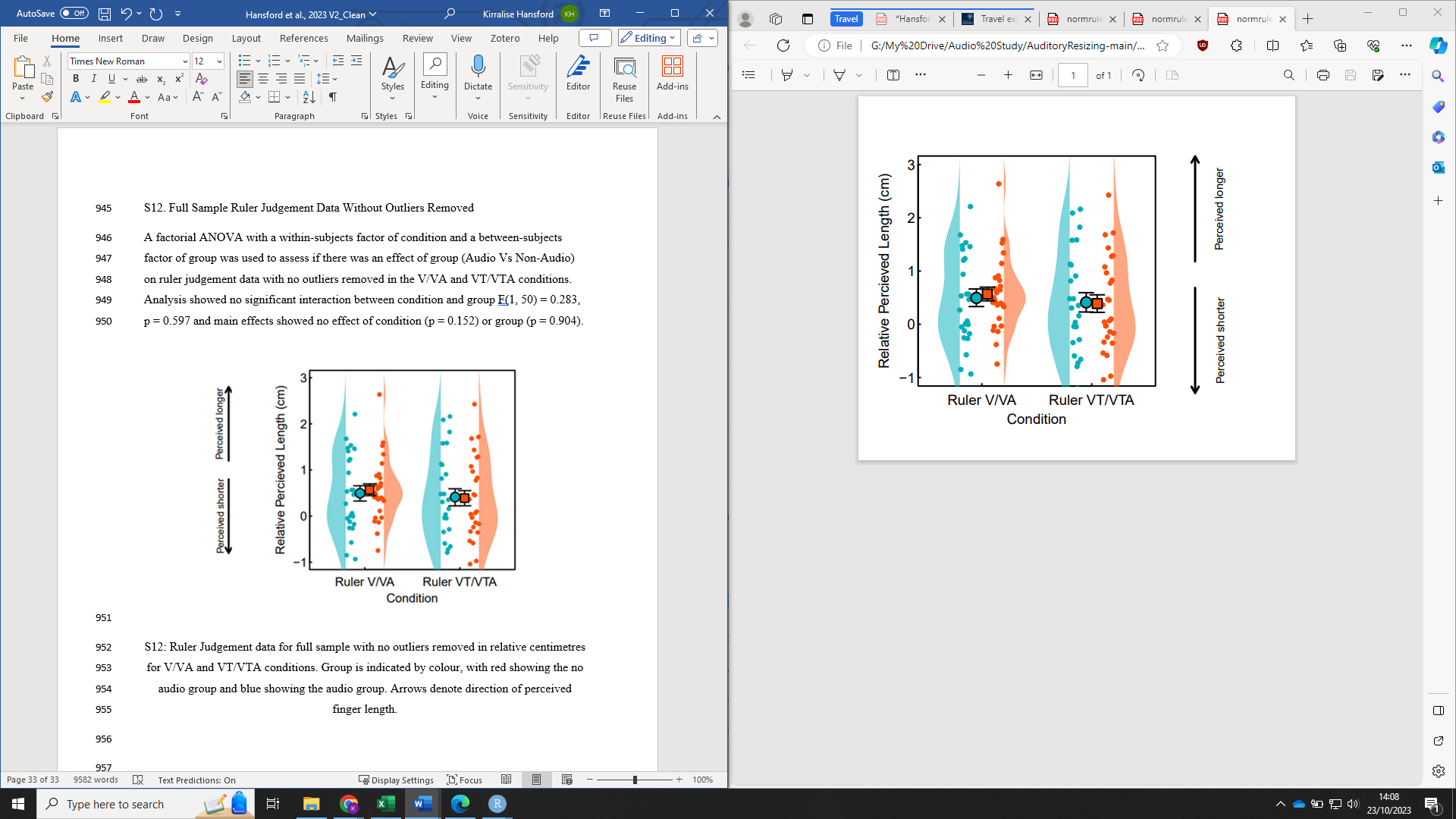
A factorial ANOVA with a within-subjects factor of condition and a between-subjects factor of group was used to assess if there was an effect of group (Audio Vs Non-Audio) on ruler judgement data with no outliers removed in the V/VA and VT/VTA conditions. Analysis showed no significant interaction between condition and group F(1, 50) = 0.283, p = 0.597 and main effects showed no effect of condition (p = 0.152) or group (p = 0.904).

S11: Ruler Judgement data for full sample in relative centimetres for V/VA and VT/VTA conditions. Group is indicated by colour, with red showing the no audio group and blue showing the audio group. Arrows denote direction of perceived finger length.

S12. Exploratory Correlation Analyses

Exploratory correlation analyses were run to assess relationships between subjective illusion score and performance-based measures of resizing illusions and can be seen in Figure 4. These were not included in our preregistration, but were suggested by a reviewer. Significance was assessed against Bonferroni correction for 4 comparisons within each correlation, at an initial alpha of .05, resulting in significance now being assessed at a revised alpha of .0125. Spearman rank correlation analyses found no significant relationships between subjective illusion score and performance on the right dot task (VA: r(42) = -0.52, p= 0.015; V: r(42) = 0.16, p=0.456; VTA: r(42) = -0.18, p= 0.437; VT: r(42) = -0.28, p= 0.203), or when comparing subjective illusion data to left dot touch data (VA: r(42) = 0.2,p= 0.381; V: r(42) = -0.071, p= 0.747; VTA: r(42) = 0.027, p= 0.908; VT: r(42) = 0.0094, p= 0.966), or comparing subjective data ruler judgement data (VA: r(42) = 0.23, p= 0.314; V: r(42) = -0.27, p= 0.214; VTA: r(42) =0.21, p= 0.353; VT: r(42) = 0.35, p= 0.098).


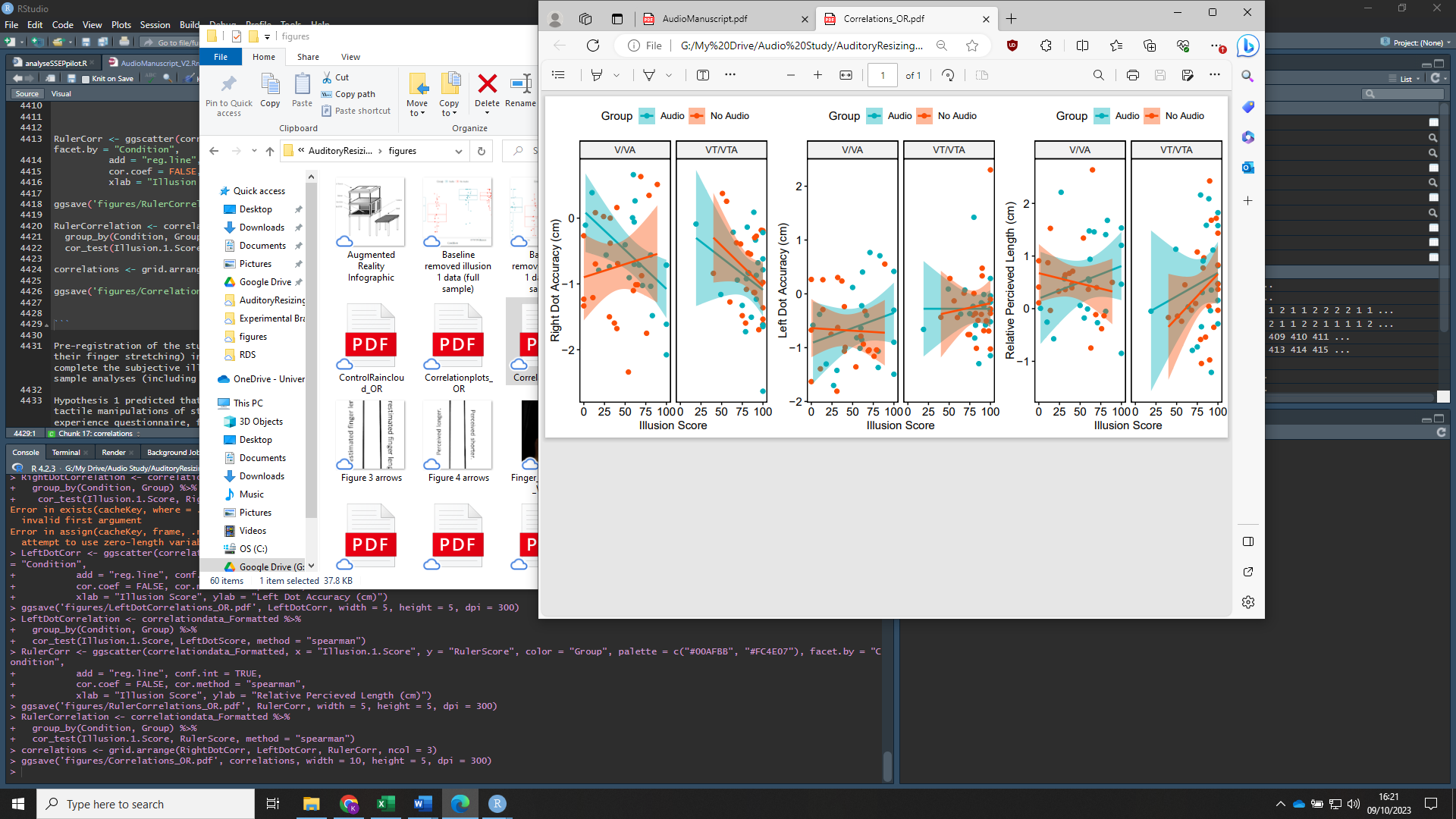


S12: Correlations between Illusion Score and Right Dot, Left Dot, and Ruler Judgement data respectively. Shading shows confidence intervals and group is indicated by colour, with red showing the no audio group and blue showing the audio group.
